# Gastroschisis Due to Maternal Exposure to Carbimazole in A Term Neonate

**DOI:** 10.7759/cureus.24808

**Published:** 2022-05-07

**Authors:** Suzan S Asfour, Mountasser M Al-Mouqdad

**Affiliations:** 1 Clinical Pharmacy Department, King Saud Medical City, Riyadh, SAU; 2 Neonatal Intensive Care Unit, King Saud Medical City, Riyadh, SAU

**Keywords:** term, neonate, hyperthyroidism, gastroschisis, carbimazole

## Abstract

Pregnant women with hyperthyroidism need careful management, which is due to the increased risk of maternal and fetal complications. However, safe and effective therapies for hyperthyroidism during pregnancy remain limited. Carbimazole is an anti-thyroid agent and has been used to treat hyperthyroidism. Gastroschisis is a congenital severe abdominal birth defect of the fetus but its etiology remains unclear. Here, we present a case of a female term neonate who developed gastroschisis after being exposed to carbimazole in uteroduring the first and half of the second trimester.

## Introduction

Pregnant women with hyperthyroidism need careful management, which is due to increased risk of maternal and fetal complications, such as fetal loss, pre-eclampsia, heart failure, thyroid storm with labor, premature labor, increased abortion rate, and low birth weight infants [1]. However, safe and effective therapies for hyperthyroidism during pregnancy remain limited. Radioactive iodine is contraindicated during pregnancy [2]. Anti-thyroid drugs (carbimazole and propylthiouracil) are teratogenic, particularly if given during the first trimester [3]. Here, we present the case of a newborn who developed gastroschisis after being exposed to low-dose carbimazole in utero.

## Case presentation

**Figure 1:**
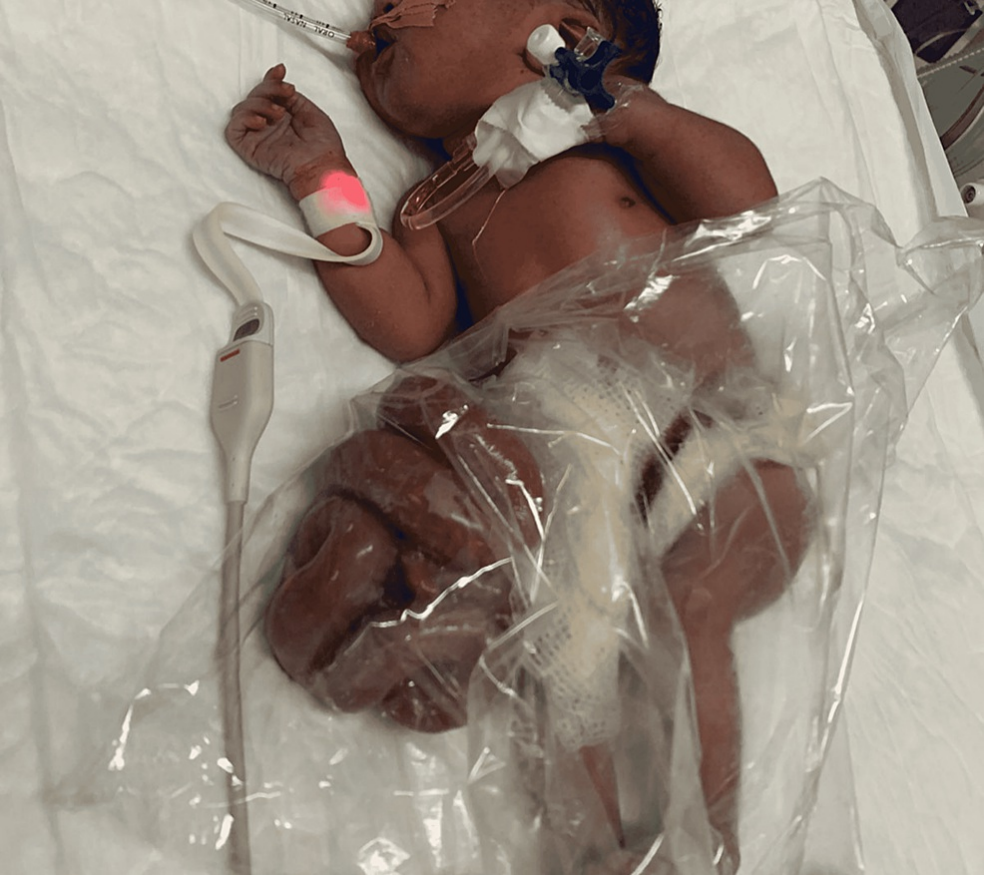
The term infant with gastroschisis

A female term neonate weighing 1900 g was delivered at home through normal spontaneous vaginal delivery; her mother was a 25-year-old woman with gravida 3 para 2. The mother had a history of hyperthyroidism with irregular antenatal care. An antenatal ultrasound scan at 9 and 20 weeks of pregnancy revealed no abnormality. She did not take any medication except carbimazole (15 mg daily) before conception and continued until 20-week gestation. Her previous two babies were normal, and according to her, she did not take any anti-thyroid medications in the last two pregnancies. She was a stay-at-home parent and had no history of radiation exposure. After two and half hours of delivery, the baby was brought to the emergency room of a tertiary care hospital. She was hypothermic and had tachypnea. A clinical examination revealed intestine evisceration from the abdomen. There were no other abnormalities discovered. The baby was intubated, connected to a mechanical ventilator, and intravenous fluid was initiated. Complete blood count, coagulation screen, and renal, liver, and bone profiles were requested; the results were normal. Ultrasound scan for kidneys was normal. Based on the clinical findings, gastroschisis and respiratory distress syndrome were diagnosed (Figure 1).

The baby was immediately shifted to the operation room for repair, revision of viability, warming, oxygenation, and regaining color. In addition to ischemic gastroschisis, intestinal malrotation was observed. However, no resection was performed at that time. The baby was administered antibiotics to rule out the early onset of sepsis. A sudden deterioration required increased oxygen, elevated serum lactate, and severe metabolic acidosis on the second day.

**Figure 2:**
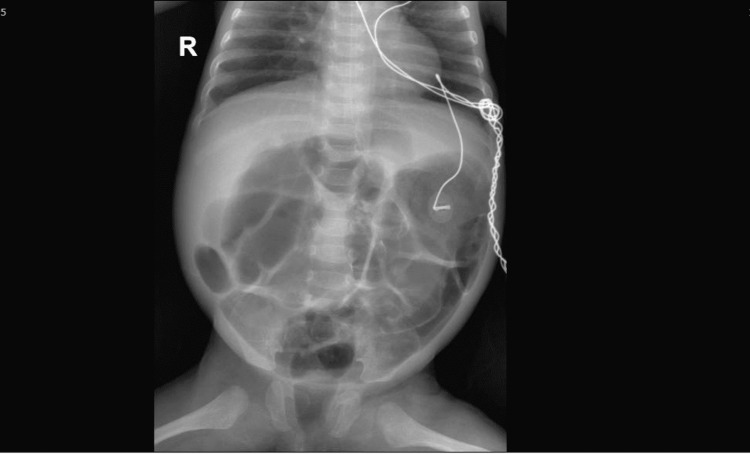
Abdominal X-ray showing abdominal distention

Besides, the baby developed a picture of necrosis versus compartment. Thus, surgical treatment by explorative laparotomy was performed. The operative findings revealed ischemia of the small bowel and end-to-end anastomosis after resecting 32 cm of the ischemic small intestine. The postoperative patient was administered total parenteral nutrition. At 13 days, the baby was extubated and shifted to the nasal cannula. At 16 days, the baby was on room air. During her follow-up in the neonatal intensive care unit, a gradual increase in abdominal distention with increased orogastric tube aspiration was observed. Due to unresolved abdominal distention, laparotomy was performed on the 35th postoperative day following the diagnosis of intestinal obstruction (Figure 2).

Besides severe distention of the small bowel, diffuse and severe adhesions between the small intestine, abdomen, and liver were detected, and adhesiolysis was performed.

Additionally, resection and anastomosis were performed with the total remaining small bowel length being 40 cm, leading to short bowel syndrome. At age 92 days, the baby was still without feeding. With a large amount of orogastric tube aspiration, explorative laparotomy with adhesiolysis was performed. In spite of this, the feeding could not be started. The baby died at 112 days because of sepsis and liver complications.

## Discussion

This report enlightens the possibility of developing gastroschisis in neonates following in-utero exposure to carbimazole in pregnant women with hyperthyroidism. Gastroschisis is a severe congenital disability in the fetus and occurs in one in 4000 births [4,5]. Ultrasound can detect gastroschisis between the 16th and 36th week of gestation [6]. The etiology of gastroschisis remains unknown with risk factors being young maternal age, a history of alcohol intake, and tobacco consumption during pregnancy [4,5]. Anti-thyroid drugs, either carbimazole or propylthiouracil, have been linked to congenital malformations in newborns during the first trimester [7]. However, little is known about the relationship between carbimazole and gastroschisis. Guignon et al. (2003) reported a case of gastroschisis after exposure to carbimazole (60 mg/day) in the first trimester followed by 20 mg/day plus levothyroxine 20 mcg/day until delivery [8]. Purnamasari et al. (2019) published a study showing the relationship between a high dose of methimazole (120 mg/day) followed by severe hypothyroidism and gastroschisis development in newborns [9]. Here, the link between in-utero carbimazole exposure and neonatal gastroschisis contributed to various observations. First, the Food and Drug Administration in pregnancy classified carbimazole as Category D due to evidence of fetal risk. Second, carbimazole will metabolize into active metabolite (methimazole), crossing the placenta [10]. Third, in addition to the previous two gastroschisis cases, four congenital abdominal wall defects had been reported and related to fetal exposure to carbimazole or methimazole in utero (two omphaloceles, one patent urachus, and one patent vitelline duct) [11]. Fourth, using the Naranjo probability scale, we discovered a possible relationship between carbimazole exposure in utero and gastroschisis in our case [12]. Our case is different from the other two cases in the following ways: first, the mother had a euthyroid state before conception. Second, she received a low dose of carbimazole (15 mg/day) until 20 weeks of gestation. Third, she did not receive levothyroxine during her pregnancy. Fourth, she remained in a euthyroid state even after carbimazole cessation. Finally, our patient died because of sepsis complications, while the previous two cases died in the first 24 hours of delivery. Thus, from this report, we recommend increasing the awareness of physicians about serious fetal complications related to using carbimazole during pregnancy. Therefore, counseling women before conception, alternative therapies if hyperthyroidism is difficult to control, and close fetal ultrasonography follow-ups are recommended.

## Conclusions

Anti-thyroid drugs, either carbimazole or propylthiouracil, have been linked to congenital malformations in newborns. Our case highlights the possibility of developing gastroschisis in neonates following in-utero exposure to carbimazole in pregnant women with hyperthyroidism. Therefore, it is recommended that pregnant women with hyperthyroidism receive preconception counseling to avoid maternal and fetal complications.
